# Intragenic and Extragenic Suppressors of Temperature Sensitive Mutations in the Replication Initiation Genes *dnaD* and *dnaB* of *Bacillus subtilis*

**DOI:** 10.1371/journal.pone.0006774

**Published:** 2009-08-26

**Authors:** Megan E. Rokop, Alan D. Grossman

**Affiliations:** Department of Biology, Massachusetts Institute of Technology, Cambridge, Massachusetts, United States of America; Cairo University, Egypt

## Abstract

**Background:**

The *Bacillus subtilis* genes *dnaD* and *dnaB* are essential for the initiation of DNA replication and are required for loading of the replicative helicase at the chromosomal origin of replication *oriC*. Wild type DnaD and DnaB interact weakly in vitro and this interaction has not been detected in vivo or in yeast two-hybrid assays.

**Methodology/Principal Findings:**

We isolated second site suppressors of the temperature sensitive phenotypes caused by one *dnaD* mutation and two different *dnaB* mutations. Five different intragenic suppressors of the *dnaD23ts* mutation were identified. One intragenic suppressor was a deletion of two amino acids in DnaD. This deletion caused increased and detectable interaction between the mutant DnaD and wild type DnaB in a yeast two-hybrid assay, similar to the increased interaction caused by a missense mutation in *dnaB* that is an extragenic suppressor of *dnaD23ts*. We isolated both intragenic and extragenic suppressors of the two *dnaBts* alleles. Some of the extragenic suppressors were informational suppressors (missense suppressors) in tRNA genes. These suppressor mutations caused a change in the anticodon of an alanine tRNA so that it would recognize the mutant codon (threonine) in *dnaB* and likely insert the wild type amino acid (alanine).

**Conclusions/Significance:**

The intragenic suppressors should provide insights into structure-function relationships in DnaD and DnaB, and interactions between DnaD and DnaB. The extragenic suppressors in the tRNA genes have important implications regarding the amount of wild type DnaB needed in the cell. Since missense suppressors are typically inefficient, these findings indicate that production of a small amount of wild type DnaB, in combination with the mutant protein, is sufficient to restore some DnaB function.

## Introduction

Initiation of DNA replication is an important event in the cell cycle. In bacteria, several proteins are required for initiation, but not elongation, of replication. DnaA is the most highly conserved replication initiation protein and is found in virtually all bacteria [1]–[3]. It binds to sequences in the chromosomal origin of replication, *oriC*, and causes melting of an AT-rich region in *oriC* creating an open complex. In addition to DnaA, the proteins needed to load the replicative helicase at the origin are also needed for replication initiation. In *Bacillus subtilis*, these include DnaD, DnaB (not to be confused with the *E. coli* replicative helicase DnaB), and DnaI. These proteins are conserved in low GC content Gram-positive bacteria and in some cases are known to be required for replication initiation {e.g., [4], [5]}. In *B. subtilis*, all are needed to load the helicase (DnaC in *B. subtilis*) at *oriC* [6], [7]. They are also needed to load helicase at stalled replication forks [8] in a process that normally requires the restart protein PriA [8]–[10]. Mutations that bypass the need for *priA* in replication restart have been described in both *E. coli* [11], [12] and *B. subtilis* [8]. In *E. coli*, these mutations are typically in the gene for the helicase loader (*E. coli dnaC*) [11], [12]. In *B. subtilis*, these mutations are in *dnaB*, part of the helicase loading machinery [6]–[8].

The *dnaD* and *dnaB* gene products are both essential proteins that interact with each other in vitro [13] and apparently in vivo [6]. DnaD also interacts with DnaA [14] and PriA [8]–[10]. In a growing population of cells, DnaB, but not DnaD, is normally found in membrane fractions of *B. subtilis* and *dnaB* is needed for enrichment of the *oriC* region in membrane fractions [6], [15]–[17]. Temperature sensitive mutations in *dnaD* and *dnaB* cause a block in replication initiation at the non-permissive temperatures. Four temperature sensitive mutations in *B. subtilis dnaB* have been described: *dnaB134ts* (also called *dnaB37ts*), *dnaB1ts*, *dnaB27ts*, and *dnaB19ts* [17]. One temperature sensitive mutation in *B. subtilis dnaD*, *dnaD23ts*, has been described [18].

Previously, we described the isolation and characterization of suppressors (temperature resistant revertants) of the temperature sensitive phenotypes caused by *dnaD23ts* and *dnaB134ts* [6]. In both selections for temperature resistant revertants, we isolated the same missense mutation in *dnaB*, *dnaBS371P*, that causes a serine to proline change at amino acid 371. That is, *dnaBS371P* is an extragenic suppressor of the temperature sensitive phenotype of *dnaD23ts* cells, and an intragenic suppressor of the temperature sensitive phenotype of *dnaB134ts* cells [6]. *dnaBS371P*, also called *dnaB75*, had been isolated previously based in its ability to bypass the need for PriA in replication restart [8]. It was isolated a third time, independently, based on its ability to suppress *dnaD23ts* [13]. The DnaBS371P mutant protein has increased affinity for DNA in vitro [7], [8]. It also detectably interacts with DnaD in a yeast two-hybrid assay [6], in contrast to the lack of detectable interaction between wild type DnaB and DnaD [6], [14], [19]. The DnaBS371P mutant protein also recruits DnaD to the membrane fraction of *B. subtilis* cells, indicating an increased interaction between these proteins in vivo [6].

In addition to the extragenic suppressor of the *dnaD23ts* mutation, three different intragneic suppressors have been described [13]. Here we describe additional intragenic suppressors of the *dnaD23ts* mutation. We found that one of the intragenic suppressors caused increased interaction between the mutant DnaD and wild type DnaB in a yeast two-hybrid assay, similar to the increased interaction between wild type DnaD and the mutant DnaBS371P [6].

We also isolated suppressors of two different *dnaBts* mutations, *dnaB19ts* and *dnaB134ts*. In both cases, intragenic and extragenic suppressors were isolated. None of the extragenic suppressors were in replication genes and most appeared to be informational suppressors.

## Materials and Methods

### Media and growth conditions

Media and growth conditions were as previously described [6]. Briefly, rich medium (LB) was used for routine growth and maintenance of *E. coli* and *B. subtilis*. Transformations were done using standard procedures [20], [21]. Antibiotics were used at the following concentrations: ampicillin (100 µg/ml); spectinomycin (100 µg/ml); chloramphenicol (5 µg/ml); and erythromycin (0.5 µg/ml) with lincomycin (12.5 µg/ml) to select for the *mls* marker.

### Strains and alleles

*E. coli* strains used for cloning and procedures used for strain constructions were as previously described [6]. *B. subtilis* strains are listed in Table 1, and are derivatives of JH642 (AG174) that contain the *trpC* and *pheA* mutations. Suppressor strains are listed in Tables 2–4. *dnaB* and *dnaD* alleles discussed here are summarized in Table 5.

**Table 1:** *B. subtilis* strains used.

Strain	Relevant genotype^1^ (notes)
BB302	Tn*917ΩHU163*::pTV21Δ2 (*cat*) (this Tn*917* insertion is linked to *dnaA*)
KI1346	*zhb83*::Tn*917*::pTV21Δ2 (*cat*) (this Tn*917* insertion is linked to *dnaB*)
KPL69	*dnaB134ts-zhb83*::Tn*917* (*mls*)
KPL73	*dnaD23ts-*Tn*917*ΩHU151 (*mls*)
KPL154	Tn*917ΩHU151*::pTV21Δ2 (*cat*) (this Tn*917* insertion is linked to *dnaD*)
KPL314	*dnaC-myc* (*spc*)
MER271	*dnaB19ts-zhb83*::Tn*917* (*mls*)

1 All strains are derived from JH642 and contain *trpC pheA* mutations.

**Table 2:** Mutations that suppress the temperature sensitive phenotype caused by *dnaDA166T* (*dnaD23ts*).

	Suppressor mutation^1^	Representative strain (#isolates)^2^
1	*dnaBS371P*	MER372 (5)
2	*dnaDΔ154-155*	MER373 (5)
3	*dnaDA138G*	MER369 (3)
4	*dnaDT148M*	MER383 (1)
5	*dnaDA166S*	MER370 (1)
6	*dnaDW188L*	MER382 (1)

1 All strains are derived from KPL73, and except for the *dnaDA166S* mutant, all contain the indicated mutation, *dnaD23ts* (*dnaDA166T*) and the linked Tn*917ΩHU151*.

2 One representative strain is indicated, along with the total number of independent isolates that were sequenced.

**Table 3 pone-0006774-t003:** Mutations that suppress the temperature sensitive phenotype caused by *dnaBK85E* (*dnaB134ts*).

	Suppressor mutation^1^	Representative strain (#isolates sequenced; total #in group)^2^
1	*dnaBS371P*	MER505 (5; 6)
2	*dnaBH65Y*	MER512 (1; 5)
3	*dnaBS151P*	MER524 (1; 6)
4	*dnaBA164V*	MER510 (2; 6)
5	*dnaBE288K*	MER517 (3; 6)
6	likely in *rrnO*	MER498^3^

1 All strains are derived from KPL69 and contain the indicated suppressor, *dnaB134ts* (*dnaBK85E*) and the linked *zhb83*::Tn*917* (*mls*).

2 One representative strain is indicated, along with the total number of independent isolates that were sequenced. In addition, the second number in parentheses indicates the total number of mutants with a phenotype indistinguishable from that of the sequenced representatives.

3 The suppressor mutation in this strain appears to be in the *rrnO* operon (which includes *trnO-ala* and *trnO-ile*). There were four suppressors in this class. The suppressor mutation in the strain indicated (MER498) was linked to the *rrnO* operon and a clone of the operon suppressed *dnaB134ts* when integrated into the genome. The suppressors in the other three strains were neither cloned nor tested for linkage. The sequence of each of the two tRNA genes in all four suppressors was wild type, indicating that the mutation, at least the one cloned, is most likely in an rRNA gene in the *rrnO* operon.

**Table 4:** Mutations that suppress the temperature sensitive phenotype caused by *dnaBA379T* (*dnaB19ts*).

	Suppressor mutation^1^	Representative strain (#isolates sequenced; total #in group)^2^
1	*dnaBT355I*	MER284 (1; 7)
2	*dnaBT366N*	MER281 (1; 5)
3	*trnO-ala* anticodon	MER280 (1; 8)
4	*trnA-ala* anticodon	MER274 (2; 4)
5	*trnB-ala* anticodon	MER283 (1; 1)

1 All strains are derived from MER271 and contain the indicated suppressor, *dnaB19ts* (*dnaBA379T*) and the linked *zhb83*::Tn*917* (*mls*).

2 One representative strain is indicated, along with the total number of independent isolates that were sequenced. In addition, the second number in parentheses indicates the total number of mutants with a phenotype indistinguishable from that of the sequenced representatives.

**Table 5:** Summary of *dnaB* and *dnaD* mutations discussed in this work.

Allele^1^ (other names)	Comments/phenotype	Reference
*dnaDA166T* (*dnaD23*)	temperature sensitive	[18]
*dnaDΔ154-155*;*A166T*	Δ154-155 suppresses A166T (DnaD23)	this work
*dnaDA138G*;*A166T*	A138G suppresses A166T	this work
*dnaDT148M*;*A166T*	Y148M suppresses A166T	this work
*dnaDA166S*	A166S isolated as a suppressor of A166T	this work
*dnaDA166T*;*W188L*	W188L suppresses A166T	this work
*dnaDA166T;L193V* (*dnaD321*)	L193V suppresses A166T	[13]
*dnaDA166T;L193I* (*dnaD325*)	L193I suppresses A166T	[13]
*dnaDΔ155-156;A166T* (*dnaD326*)	Δ155-156 suppresses A166T	[13]
*dnaBA379T* (*dnaB19*)	temperature sensitive	[17]
*dnaBK85E* (*dnaB134*; *dnaB37*)	temperature sensitive	[17]
*dnaBS371P* (*dnaB75*)	suppresses: *dnaDA166T* (*dnaD23*), *dnaBK85E* (*dnaB134*), and Δ*priA*	[6], [8], [18]
*dnaBH65Y*;*K85E*	H65Y suppresses K85E (DnaB134)	this work
*dnaBK85E*;*S151P*	S151P suppresses K85E	this work
*dnaBK85E*;*A164V*	A164V suppresses K85E	this work
*dnaBK85E*;*E288K*	E288K suppresses K85E	this work
*dnaBK85E*;*S371P*	S371P suppresses K85E	this work
*dnaBT355I*;*A379T*	T355I suppresses A379T (DnaB19)	this work
*dnaBT366N*;*A379T*	T366N suppresses A379T	this work

1 The mutant allele is named using the convention indicating the wild type amino acid, the codon number and the mutant amino acid. Intragenic suppressors contain the original mutation (*dnaBK85E*, *dnaBA379T*, or *dnaDA166T*) as indicated.

DnaD is 232 amino acids and *dnaD23ts* causes an alanine to threonine change at amino acid 166 (A166T) [18]. DnaB is 472 amino acids and *dnaB19ts* causes an alanine to threonine change at amino acid 379 (A379T) and *dnaB134ts* (a.k.a., *dnaB37*) causes a lysine to glutamic acid change at amino acid 85 (K85E) [17]. All strains containing temperature sensitive alleles were grown at 30°C.

### Isolation of spontaneous suppressor mutations

Spontaneous suppressors were isolated by plating approximately 10^7^ or 10^8^ *dnaD23ts* (KPL73), *dnaB19ts* (MER271), or *dnaB134ts* (KPL69) cells (grown in LB medium at 30°C) onto LB plates and incubating overnight at either 45°C or 48°C, essentially as described [6].

### EMS mutagenesis

We also isolated suppressors of *dnaB19ts* following mutagenesis with ethyl methanesulfonate (EMS). For the mutagenesis, *dnaB19ts* (MER271) cells were grown in LB medium at 30°C to an OD_600_ = 0.4. Cells were washed twice with LB and resuspended to an OD_600_ = 1.0. Seven independent pools of cells were mutagenized in LB with 1.2% EMS (Sigma) for 30 minutes at 30°C and then washed twice with LB. Cells were grown for four generations at 30°C in LB without mutagen for recovery, and plated on LB at 45°C or 48°C for selection of temperature resistant suppressors.

### Classification and mapping of suppressor mutations

Suppressor mutations were classified and mapped essentially as described [6]. Briefly, colonies of suppressor strains were purified three times and grouped by growth phenotypes on LB plates incubated at 30°, 37°, 42°, 45°, 48°, and 52°. Isolates that were indistinguishable from each other under all conditions were grouped together. Representatives from each group were tested for linkage to replication genes *dnaA*, *dnaB*, *dnaC* (helicase), and *dnaD* using antibiotic-resistant markers linked to the wild-type allele of each gene. For *dnaA*, *dnaB*, and *dnaD*, a Tn*917* insertion containing the chloramphenicol resistance gene (*cat*) from the plasmid pTV21Δ2 linked to each gene was used as a selectable marker [22], [23]. Tn*917ΩHU163* is linked to *dnaA*, *zhb-83*::Tn*917* is linked to *dnaB*, and Tn*917ΩHU151* is linked to *dnaD* [23], [24]. Testing for linkage to *dnaC* (helicase) was done with the spectinomycin resistance gene associated with a *dnaC-myc* fusion from strain KPL314 [6]. Suppressor alleles linked to *dnaD* and *dnaB* were amplified by PCR and the products either sequenced directly or cloned and then sequenced. For suppressors thought to be in alanine tRNA genes, the relevant tRNA genes were amplified by PCR and either sequenced directly or cloned and sequenced. The *rrnO* locus was cloned into the integrative vector pGEM*cat* [25].

### Two-hybrid analysis

*dnaD* alleles *dnaD23Δ154-155, dnaD23W188L, dnaD23T148M*, and *dnaD23A138G* were amplified by PCR from the appropriate chromosomal DNA and inserted into the vector pGBDU-C3 to create in frame fusions of DnaD23Δ154-155, DnaD23W188L, DnaD23T148M, and DnaD23A138G to the DNA binding domain (DBD) of the Gal4 transcription factor [26]. The resulting plasmids, pMR81 (encoding DBD-DnaD23Δ154-155), pMR82 (encoding DBD-DnaD23W188L), pMR83 (encoding DBD-DnaD23T148M), and pMR80 (encoding DBD-DnaD23A138G) were individually co-transformed with pMR58 (encoding AD-DnaB) into the yeast strain DWY112, and the yeast two-hybrid analysis was performed as described previously [6].

## Results and Discussion

### Rationale

DnaD and DnaB are involved in multiple protein and DNA interactions. The existence of mutations in *dnaB* that cause altered interaction between DnaB and DnaD indicate that these proteins are likely targets for regulatory factors. One approach to study DnaB and DnaD, and to possibly identify regulators of their functions, is to identify second site mutations that suppress phenotypes caused by *dnaB* and *dnaD* mutations. In the case of intragenic second site suppressors, this approach also has the potential to provide structure-function insights. To these ends, we isolated and characterized second site suppressors of a temperature sensitive mutation in *dnaD* and two different temperature sensitive mutations in *dnaB*. Temperature resistant revertants were isolated and characterized phenotypically to determine which were most likely to be second site suppressors and not simple back mutations. Isolates that were more temperature resistant than the original mutant, but also more temperature sensitive than the true wild type were chosen for further analyses. Most of the second site suppressors were intragenic and the *dnaD* and *dnaB* alleles discussed in this work are listed in Table 5.

### Isolation of suppressors of the temperature sensitive phenotype caused by *dnaD23ts*

We isolated 25 independent spontaneous suppressors (temperature resistant revertants) of the temperature sensitive phenotype caused by the *dnaD23ts* mutation (summarized in Table 2). The *dnaD23ts* allele (*dnaDA166T*) causes an alanine to threonine change at amino acid 166 of DnaD [18]. Five of the 25 suppressors were extragenic and all contained the *dnaBS371P* mutation (Table 2, line 1) described previously [6], [13].

The remaining 20 apparently intragenic suppressor mutants fell into six classes based on differences in growth and colony phenotypes on LB plates at a series of temperatures. The phenotypes of one group of 9 independent revertants were indistinguishable from those of wild type *dnaD* ^+^ cells. They grew as well as wild-type through the full range of temperatures at which wild-type grows. We chose not to characterize these because it was not clear that they represented second site suppressors.

The remaining 11 suppressor mutants fell into 5 groups. All of these suppressor mutations were sequenced and found to be in *dnaD* (Table 2). They include: *dnaDT148M* and *dnaDW188L*, each isolated once and *dnaDA138G* isolated three times independently. Another group of intragenic suppressors consisted of one isolate, which contained the suppressor mutation *dnaDA166S*. The original *dnaD23ts* mutation causes an alanine to threonine change at amino acid 166 [18], and thus this suppressor mutation, which causes an alanine to serine change at amino acid 166, suppresses the temperature sensitivity caused by *dnaD23ts* by changing the threonine at position 166 of the mutant DnaD23 to a serine.

The final group of *dnaD23ts* suppressors consisted of five isolates, all of which contained the identical intragenic suppressor mutation. This mutation is a deletion of six base pairs from a region that contains a repeat of the five base pair sequence 5′-CAGGA. This six base pair deletion (between the repeated sequence) leads to deletion of two amino acids in DnaD, amino acids 154 (asp) and 155 (gln). We refer to the allele of *dnaD* that contains both this deletion and the original mutation that causes the temperature sensitivity as *dnaD23Δ154-155* (Table 2, line 2).

Three intragenic suppressors of *dnaD23ts* were described previously [13]. These include: *dnaD321* causing a leucine to valine change at condon 193 (*dnaDL193V*), *dnaD325* causing a leucine to isoleucine change also at amino acid 193 (*dnaDL193I*), and *dnaD326* causing a deletion of glutamine and aspartate at amino acids 155 and 156 (*dnaDΔ155-156*). All three of these suppressors are different from the alleles described here, although the 2 amino acid deletions are remarkably similar. That we isolated different alleles in our experiments indicates that none of the selections have been saturated.

### *dnaD23Δ154-155* allows for the detection of the physical interaction between DnaD and DnaB

We previously found that the only extragenic suppressor isolated in the *dnaDts* selection, *dnaBS371P*, allowed for detection of an interaction between DnaB and DnaD in a yeast two-hybrid assay [6]. Just as *dnaBS371P* is located at the region of DnaB that is similar to a family of phage replication proteins [6], the five intragenic suppressors of *dnaDts* were also located in the phage homology region of DnaD. We used yeast two-hybrid analysis [26] to test whether any of the *dnaDts* intragenic suppressors allowed for a detectable interaction between mutant DnaD and wild-type DnaB.

**Figure 1:**
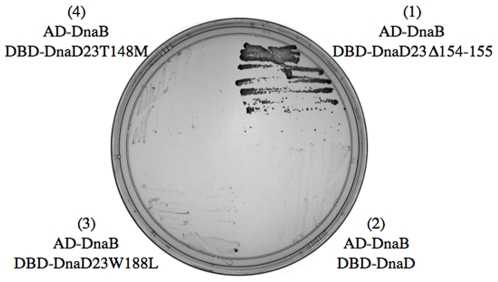
DnaD23Δ154-155 interacts with DnaB. Yeast two-hybrid analysis was used to examine physical interactions between DnaB fused to the activation domain (AD) and wild type and mutant forms of DnaD fused to the DNA binding domain (DBD) from the Gal4 transcription factor. A physical interaction activates the expression of ADE2, allowing for growth on medium lacking adenine. Plate section 1, upper right (AD-DnaB, DBD-DnaD23Δ154-155), shows that DnaB and DnaD23Δ154-155 interact. Plate section 2, lower right (AD-DnaB, DBD-DnaD), confirms previous results that DnaB and DnaD do not detectably interact in the two-hybrid assay. Plate sections 3, lower left (AD-DnaB, DBD-DnaD23W188L), and section 4, upper left (AD-DnaB, DBD-DnaD23T148M), show that DnaB does not detectably interact with DnaD23W188L or DnaD23T148M in this assay.

We found that DnaD23Δ154-155 and DnaB interact in the yeast two-hybrid assay. We fused DnaB to the activation domain (AD) and DnaD23Δ154-155 to the DNA binding domain (DBD) of the yeast transcription factor Gal4. In this system, a physical interaction between the Gal4 AD and DBD domains will drive expression of ADE2, thereby allowing for growth of the yeast on medium lacking adenine [26]. We found that yeast expressing AD-DnaB and DBD-DnaD23Δ154-155 grew on medium lacking adenine, indicating that DnaD23Δ154-155 and DnaB physically interact (Figure 1). Although we have not tested this directly, we suspect that in *B. subtilis*, the *dnaD23Δ154-155* mutation allows for increased or unregulated interactions to occur between DnaB and DnaD, just as *dnaBS371P* does [6].

In contrast to the yeast two-hybrid results indicating increased interaction between DnaD23Δ154-155 and DnaB, we did not detect physical interactions between the other mutant forms of DnaD and DnaB. Wild-type DnaB did not detectably interact in the two-hybrid assay with either DnaD23T148M or DnaD23W188L (Figure 1). The intragenic suppressor *dnaD23A138G* did not yield reproducible results, and the intragenic suppressor *dnaDA166S* was not tested.

### Possible mechanisms of intragenic suppression of *dnaD23ts*

There are multiple mechanisms by which intragenic suppressors could partly restore function to the DnaDA166T (DnaD23ts) mutant protein. Wild type DnaD is an oligomer [27], [28], binds to and can remodel DNA [9], [13], [28]-[32], and forms a scaffold on DNA [28], [29]. It also interacts with DnaA [14] and DnaB [6], [9], [13]. DnaD has two domains with different functions. The N-terminal domain (amino acids 1-128) has oligomerization activity and the C-terminal domain (aa 129–232) has DNA binding activity and DNA-induced oligomerization activity [28 activities].

The DnaDA166T (DnaD23ts) mutant protein has decreased binding to ssDNA in vitro, and decreased cooperativity of binding [9], [13]. The mutation is in the C-terminal DNA binding domain of DnaD. In vivo, the mutant protein is unstable, but this instability does not seem to be the primary cause of the temperature sensitive phenotype [13]. This conclusion is based on the finding that increased amounts of the mutant protein expressed from a plasmid do not suppress the temperature sensitive phenotype and the previously described intragenic suppressors did not seem to function simply by increasing stability of the mutant protein [13].

One of the intragenic suppressors, DnaD23Δ154-155, caused increased interaction with DnaB, possibly contributing to the mechanism of suppression. The loss of these two residues likely uncovers an otherwise hidden DnaB binding site. This phenotype is similar to that caused by the extragenic suppressor of *dnaD23ts* that is in *dnaB*. This extragenic suppressor, *dnaBS371P*, causes increased interaction with DnaD [6]. It also causes DnaB to have increased affinity for DNA [7], [8]. It is not yet known if the DnaDΔ154-155 mutant has increased DNA binding, but the mutation is in the domain that binds DNA. It is possible that the other intragenic suppressors of *dnaD23ts* restore the normally cooperative DNA binding and/or cause increased interaction with DnaB, although any potential increase in interaction with DnaB was not detected in a yeast two-hybrid assay.

It is also possible, although probably less likely, that the suppressors compensate for the defect caused by *dnaDA166T* (*dnaD23ts*) by altering some other aspect of DnaD function. For example, the suppressors could alter the oligomeric state of DnaD, affect interaction with DnaA, or possibly make DnaD less sensitive to uncharacterized negative regulatory factors affecting replication initiation. The suppressors are not in the N-terminal region of DnaD that contains the main oligomerization activity (aa 1–128) [28]. However, the C-terminal domain of DnaD has a DNA-stimulated oligomerization activity [28], making a possible effect on oligomerization plausible. Likewise, the suppressor mutations are not in the region of DnaD (aa 1–140) that is known to interact with DnaA [14]. However, it is not known if the C-terminal region (aa 141–232) contributes to the interaction with DnaA, nor is it known if there are residues in this region that normally reduce the interaction between DnaD and DnaA. Of course, these possibilities are not mutually exclusive and the mechanisms of suppression could involve multiple activities of DnaD and be different for different alleles.

### Intragenic suppressors of *dnaB134ts* and *dnaB19ts* mutations

We isolated 41 independent spontaneous suppressors of *dnaB134ts* (*dnaBK85E*). In addition, we isolated a total of 31 independent suppressors of *dnaB19ts* (*dnaBA379T*), some spontaneous and some following EMS mutagenesis (Materials and Methods). Suppressor strains were grouped based on growth and colony phenotypes at different temperatures and representatives of each phenotypic group were tested for linkage to different replication genes, as described above. Then, representative suppressor alleles from different linkage and phenotypic groups were sequenced (Tables 3, 4). There were 6 suppressors of *dnaB19ts* that were phenotypically indistinguishable from wild type. One of these was sequenced and was a back mutation restoring *dnaB* ^+^ and the others were not characterized further. There were 5 suppressors of *dnaB134ts* that were phenotypically indistinguishable from wild type. Since it was not clear that these were second site suppressors, none were characterized further.

#### *dnaBS371P* suppresses *dnaB134ts*

As reported previously, *dnaBS371P* is an intragenic suppressor of *dnaB134ts* (*dnaBK85E*) [6]. This suppressor allele was present in five independent isolates that were sequenced. A sixth suppressor strain isolated in this selection had the same phenotype as these five and likely also contains this same mutation (Table 3, line 1).

#### Other intragenic suppressors of *dnaB134ts*

The remaining intragenic suppressors of *dnaB134ts* fell into four groups based on growth and colony phenotypes at a series of temperatures. All groups contained members that were linked to *dnaB* and the sequence of at least one representative from each group was determined (Table 3, lines 2–5). One class contained six isolates, two of which were sequenced and each had the suppressor mutation, *dnaBA164V*. Another group contained five isolates, one of which was sequenced and had the suppressor mutation *dnaBH65Y*. Another group contained six isolates, three of which were sequenced. Each had the suppressor mutation *dnaBE288K*. The final group contained six isolates, one of which was sequenced and found to contain the suppressor mutation *dnaBS151P*.

#### Intragenic suppressors of *dnaB19ts*

There were two groups of intragenic suppressors of *dnaB19ts* (*dnaBA379T*) (Table 4, lines 1–2). One group contained five isolates, one of which was sequenced and found to contain the suppressor mutation *dnaBT366N*. The other group contained seven isolates, one of which was sequenced and found to contain the suppressor mutation *dnaBT355I*. *dnaBS371P* was not isolated as a suppressor of *dnaB19ts*.

### Possible mechanisms of intragenic suppression of *dnaB134ts* and *dnaB19ts*

As with *dnaD*, there are multiple mechanisms by which the intragenic suppressors could partly restore function to DnaBK85E (DnaB134ts) or DnaBA379T (DnaB19ts). Little is known about the mechanistic defects caused by these *dnaB* mutations, so it is difficult to know if the suppressors restore normal function or compensate for decreased function by increasing some other aspect of DnaB function. Wild type DnaB is found in membrane fractions of cells and is involved in the enrichment of *oriC* in membrane fractions [6], [15]–[17], although it is not itself an integral membrane protein as it is washed out of membrane fractions with high salt (data not shown). DnaB is a tetramer [9], [31], [33] and binds to and remodels DNA [31]. In addition, it interacts with DnaD [6], [13] and DnaI [7]. Presumably, most, if not all, of these properties are important for DnaB function in replication initiation. The intragenic suppressors could stimulate one or more of these functions or an as yet uncharacterized property of DnaB. Since DnaBS371P (DnaB75) has increased DNA binding [7] and increased interaction with DnaD [6], [13], and is an intragenic suppressor of *dnaB134ts*, it is plausible that some of the other intragenic suppressors could work similarly.

### Extragenic suppressors of *dnaB19ts* and *dnaB134ts* mutations

#### Informational suppressors of *dnaB19ts*

Three classes of suppressors of *dnaB19ts* were extragenic, as they were not linked to *dnaB*. All of these mutations caused allele-specific suppression and the mutants formed very small colonies on LB plates. Cells appeared normal in phase contrast microscopy (data not shown). Suppressors from one of these groups were linked to Tn*917ΩHU163* (near *dnaA*), but did not appear to be in *dnaA*. A ribosomal RNA and tRNA gene cluster (*rrnO*) is also linked to this transposon, and based on the growth phenotype and linkage, we suspected that the suppressors might be informational. We PCR-amplified and sequenced *trnO-ala* from the *rrnO* operon of one of the suppressor mutants and found a mutation in the tRNA-ala gene that changes the alanine anti-codon to one that will recognize a threonine codon (Table 4, line 3). That is, the mutant tRNA-ala gene contained the exact missense mutation that should allow the alanine tRNA to read the mutant codon in the *dnaB19ts* mRNA and insert the wild type alanine in place of the mutant threonine.

Based on the finding that a mutation in *trnO-ala* suppressed the temperature sensitive phenotype caused by *dnaBA379T*, we decided to sequence the tRNA-ala genes from at least one representative of the other two groups of suppressors with similar phenotypes. There are six tRNA-ala genes (including *trnO-ala*). We found that, in the three additional mutants that were characterized, each had a mutation that changes the anti-codon from recognizing an alanine codon to recognizing a threonine codon. In total, two sequenced isolates were in *trnA-ala*, and one each was in *trnB-ala* and *trnO-ala* (Table 4, lines 3–5).

#### Extragenic suppressors of *dnaB134ts* are probably in tRNA or rRNA genes

Two groups of the *dnaB134ts* (*dnaBK85E*) suppressors were not linked to *dnaB* and thus were extragenic. These caused phenotypes similar to those of the suppressors of *dnaB19ts* that were in tRNA genes. That is, all of the extragenic suppressors of *dnaB134ts* were allele-specific and caused slow growth. One group of suppressors (Table 3, line 6) contained a mutation that was linked to Tn*917ΩHU163* (near the *rrnO-trnO* operon) and suppressors in the other group were not linked to the *rrnO* operon and were not characterized further.

We cloned the *rrnO* operon from one suppressor mutant into the integration vector pGEM*cat*. When integrated into the chromosome of a *dnaB134ts* (*dnaBK85E*) mutant, this clone was able to suppress the temperature sensitive phenotype, indicating that it contained the suppressor mutation. We sequenced the two tRNA genes in the *rrnO* operon, *trnO-ala* and *trnO-ile*, from all four suppressors in this class, i.e., the cloned *rrnO* operon and the other three that were not cloned. In all four suppressors, *trnO-ala* and *trnO-ile* were wild type. This was not completely surprising since the mutation in *dnaB134ts* is in a lysine codon (changing it to glutamate) and not in an alanine or isoleucine codon.

These extragenic suppressors of *dnaB134ts* were not likely to be in any regulatory factor affecting replication initiation. Rather, we suspect that, while not in *trnO-ala* or *trnO-ile*, they might still be informational suppressors, and at least the one we cloned is likely to be in one of the rRNA genes of the *rrnO* operon (Table 3, line 6). Informational suppressors are most common in tRNA genes and the most widely known are nonsense suppressors. However, mutations in ribosomal components that affect translational fidelity and cause informational suppression have been known for a long time [34]–[36]. These can be in ribosomal protein genes or in rRNA genes [37], [38]. We suspect that the suppressor of *dnaB134ts* that appears to be in the *rrnO* cluster, but that is not in either of the tRNA genes in the *rrnO* operon, is in the 16S rRNA gene and might be causing translational misreading, thereby allowing for synthesis of some wild type DnaB protein.

### Perspectives

The isolation of temperature resistant revertants of *dnaDts* and *dnaBts* mutants did not result in the identification of new regulators of DNA replication. Most of the revertants were intragenic and some were allele-specific informational suppressors. The nature of these mutations have functional implications for DnaB and DnaD.

The isolation of mutations in tRNA-ala genes that are informational suppressors of the temperature sensitive phenotype caused by the *dnaB19ts* mutation has some interesting implications regarding DnaB function. The efficiency of suppressor tRNAs that suppress missense mutations is generally very low [39]. This low frequency would result in only a small amount of wild type DnaB in the cell, and most of the protein would still be the mutant DnaBA379T (DnaB19ts). The finding that alanine tRNA mutations are able to suppress the temperature sensitivity of *dnaB19ts* cells implies that the amount of wild-type DnaB necessary for the cell is much lower than the amount of DnaB found in wild-type cells. It may be that there is excess DnaB in wild-type cells, and thus decreasing the concentration of functional DnaB is not harmful to cells. In addition, since DnaB is a tetramer, it is possible that mixed tetramers are functional and perhaps only a single wild type protein in a tetramer is sufficient for function and restoration of temperature resistant growth to *dnaB19ts* cells. We have not tested these possibilities.

Intragenic suppressors of *dnaD23ts* that increase interaction with DnaB, and mutations in *dnaB* that increase interaction with DnaD and suppress *dnaD23ts*, together indicate that increased interaction between these proteins might be an important part of suppression. Perhaps DnaB and DnaD naturally interact in vivo, as indicated by weak interactions in vitro. The mutations that increase this interaction could either strengthen the interaction or increase the frequency of interaction, perhaps by bypassing a normal regulatory step. The increased interaction could restore a function defective in the mutant protein or enhance a different function, thereby compensating for the defect in the mutant protein. The mechanisms by which these mutations affect DnaD and DnaB should become clearer with more structural analyses of these essential replication initiation proteins.
